# The Treatment of Enlarged Prostate

**Published:** 1902-02-22

**Authors:** 


### Hospital Clinics and Medical Progress.

### THE TREATMENT OF ENLARGED PROSTATE.

In a recent clinical lecture, Mr. Hurry Fenwick [1] considered the question of the best form of operative treatment for the cure of "enlarged" prostate. There are five different procedures at present in favour with the profession in the management of these cases:—(1) Resection of one or both vasa deferentia (vasectomy). (2) Removal of one or both testes (orchidectomy). (3) Division of the prostate at the orifice of the bladder through a perineal opening (perineal prostatotomy). (4) Division of the prostate at the orifice of the bladder by an electro-cautery knife introduced along the urethra (Bottini). (5) Removal or enucleation of the obstructing parts of the prostate (perineal or suprapubic prostatectomy). It is important, however, to exercise judgment in the choice of operation, and the belief which some seem to have gathered from recent discussions on the subject of suprapubic prostatectomy to the effect that the true and only surgery of the enlarged prostate consists in scooping it out bodily from its capsule, just as one peels an orange, is, he said, an error. Only a certain proportion of cases are suitable for enucleation. First, then, as to vasectomy or orchidectomy: these operations cause atrophy of the essentially sexual portion of the prostate. The relief given by these operations is due not to the reduction in size of the prostate gland as a whole, but to the atrophic changes in the immediate neighbourhood of the prostatic urethra and the orifice of the bladder. Hence "if the lobes of the prostate are flat, and feel fibrous, if the sulcus is marked by a deep trough, and the function of the testes is probably in abeyance, neither vasectomy nor orchidectomy will be of much use in relieving the patient of his catheter. But if the prostate be elastic and its lobes project and its sulcus be normal, bilateral vasectomy or castration will give relief." All hard tough prostates in which the interlobular sulcus is filled up by a ridge of firm tissue, are unsuitable for either of these operations.

But supposing the "vim" of the patient admits of a cutting operation, the object of such an operation is to remove the obstruction—and the form of the obstruction varies. There are three forms of obstruction which will embrace most of the cases. In the first the one and only obstruction is in the form of a projecting "bar" or "collar" at the orifice of the bladder, and in this the prostatic urethra is usually wide. In the second form the only obstruction consists in a median lobe which overlies the orifice of the bladder, the prostatic urethra being in this case also wide enough for free urination. The third form embraces the very large adenomatous prostates in which intra-vesical lobes not only project over and obstruct the urethral orifice, but additional tumours also bulge into the urethral canal and render that channel narrow, distorted, and especially difficult to catheterise. Now here we come to a point where Mr. Hurry Fenwick clears up much of the misunderstanding which seems to have existed in many minds as to the relation of the capsule of the prostate to prostatectomy. We have been told by some that the entire prostate can be shelled out. Mr. Hurry Fenwick, however, shows how huge and intricate are the venous meshes which lie on the outer side of the capsule in the older prostates; how intimately they are connected with it, and how they interpenetrate its outer layers; and thus how deadly would be the hæmorrhage were they interfered with, or were the capsule of the gland split or perforated. Hence, in the operation of enucleation the finger and lever are kept religiously *inside the capsule* and pressure is exerted as much as possible upon the adenomatous masses to be enucleated. Lastly he shows how densely adherent is the prostatic tissue, in the adult or in the "tough" prostate, to the inside of the prostatic capsule. "You cannot dissect it off, because the web and woof of the prostatic tissue is derived from the capsule itself." How then are we to explain the claim made that the whole prostate is enucleated? Those who choose to read between the lines of the following paragraph will probably attribute to Mr. Fenwick the opinion that what is removed is not, in a literal sense, the prostate which, by the time enucleation has become desirable, has long since been so atrophied by pressure that there is but little of it left, but the adenomata by which this pressure has been produced. Mr. Fenwick says: "But take this old adenomatous prostate and you will see that the tremendous expansion of adenomatous growth has altered this intimate relation. What is left of the unchanged prostatic tissue only exists in patches as fine lines next the capsule, and the new material can be shelled out piecemeal or as a whole with ease and without interfering with the integrity of the capsule. Thus," he adds, "Mr. Freyer grasped the idea that in enucleating adenomatous masses we were practically often emptying the capsule of the prostate, and that it was often wise to do so."

Coming now to the question of treatment. If, on examination, the condition found falls under the description of the "bar" or "collar" group, the lobes being dense and fibrous and inelastic with an obliterated sulcus, we have to do with a gland the lobes of which will not shell out nor even tear out, and in such a case perineal prostatotomy may be done. If, on the other hand, the case be one of median lobe enlargement, suprapubic cystotomy may be done, the median lobe may be removed, the stump being, if possible, covered by flaps of mucous membrane, and at the same time the condition of the rest of the prostate may be fully investigated, and if found adenomatous, the enucleation may be carried further; while lastly, if the case be one of adenomatous growths ripe for enucleation, enucleation may be performed. But it is to be borne in mind that, take the cases altogether, only about 50 per cent., or thereabouts, of the prostates one encounters can be shelled out of their capsules. "To put the matter in a nutshell. There are two essential groups to consider—the small, tough, flat, unshellable prostate, which generally has a filled-up sulcus . . . . and the large, soft, elastic, projecting shellable prostate, which has a fine line of sulcus. Between these two forms are, of course, many gradations in elasticity." The "ripeness" for

B

removal is to be judged by the tension of the capsule and the appreciation of this must depend on the tactile experience of the forefinger.

[1] Practitioner, February.

